# Beyond repair - family and community reintegration after obstetric fistula surgery: study protocol

**DOI:** 10.1186/s12978-015-0100-1

**Published:** 2015-12-18

**Authors:** Josaphat Byamugisha, Alison El Ayadi, Susan Obore, Haruna Mwanje, Othman Kakaire, Justus Barageine, Felicia Lester, Elizabeth Butrick, Abner Korn, Hadija Nalubwama, Sharon Knight, Suellen Miller

**Affiliations:** Department of Obstetrics and Gynecology, Makerere University College of Health Sciences, Kampala, Uganda; Mulago National Referral and Teaching Hospital, Kampala, Uganda; Department of Obstetrics, Gynecology and Reproductive Sciences, Bixby Center for Global Reproductive Health, University of California San Francisco, California, USA

**Keywords:** Obstetric fistula, Social reintegration, Beyond repair, Maternal morbidity, Obstructed labor, Measurement tool, Uganda

## Abstract

**Background:**

Obstetric fistula is a debilitating birth injury that affects an estimated 2–3 million women globally, most in sub-Saharan Africa and Asia. The urinary and/or fecal incontinence associated with fistula affects women physically, psychologically and socioeconomically. Surgical management of fistula is available with clinical success rates ranging from 65–95 %. Previous research on fistula repair outcomes has focused primarily on clinical outcomes without considering the broader goal of successful reintegration into family and community.

The objectives for this study are to understand the process of family and community reintegration post fistula surgery and develop a measurement tool to assess long-term success of post-surgical family and community reintegration.

**Methods:**

This study is an exploratory sequential mixed-methods design including a preliminary qualitative component comprising in-depth interviews and focus group discussions to explore reintegration to family and community after fistula surgery. These results will be used to develop a reintegration tool, and the tool will be validated within a small longitudinal cohort (*n* = 60) that will follow women for 12 months after obstetric fistula surgery. Medical record abstraction will be conducted for patients managed within the fistula unit. Ethical approval for the study has been granted.

**Discussion:**

This study will provide information regarding the success of family and community reintegration among women returning home after obstetric fistula surgery. The clinical and research community can utilize the standardized measurement tool in future studies of this patient population.

## Background

Obstetric fistula (OF) is a debilitating birth injury that may affect as many as 2 to 3 million women globally, most in sub-Saharan Africa and Asia [1]. Recent analyses suggest this statistic may be an overestimate, supporting a lifetime prevalence of 3 per 1000 women of reproductive age in sub-Saharan Africa [2], and an estimated 6000 new cases per year across sub-Saharan Africa and south Asia [3]. However, while truly robust data on the epidemiology of OF simply do not yet exist [3–5], the available facility and community data sources support OF as an important public health concern. OF is primarily due to prolonged obstructed labor from cephalopelvic disproportion or malpresentation in the absence or delay in access of comprehensive emergency obstetric care. Previous literature has described the devastating physical, psychological and social sequelae endured by women with obstetric fistula [6–11]. Women with obstetric fistula suffer from urinary and/or fecal incontinence. As a result, they develop infections, vaginal and genital ulcerations, and may also suffer from nerve damage, an additional sequelae of the prolonged obstructed labor, including foot drop which makes walking without assistance difficult. Many also develop secondary infertility. The physical effects of obstetric fistula overwhelm the marginalized women suffering from this condition, yet the psychological effects can be even more devastating. Constantly leaking urine and feces, obstetric fistula sufferers are stigmatized and ashamed of their offensive smell and inability to be clean which results in isolation and abandonment from families and communities [12, 13].

Surgery is available to treat obstetric fistula, and clinical success rates range from 65–95 % [14, 15]. Efforts by local, national and international agencies have dramatically improved women’s access to obstetric fistula surgery over the past decade. However, previous research on the success of fistula surgery is largely limited to the short-term clinical outcomes of surgery, largely assuming concomitant positive social and emotional effects with successful surgery [7, 16, 17]. Clinical outcomes are very important, but they may not represent the comprehensive changes necessary to restore women’s quality of life. Continence may not be achieved even with a successful fistula closure if the urethra is not functional or the continence mechanism has been destroyed. As many as one-third of women experience persistent incontinence post-surgery [17]. Little attention has been paid to success of the surgery from the woman’s perspective, including her experience reintegrating into family and community life. Furthermore, returning to families and communities comes with an expectation of change, or return to certain social roles. About 90 % of women experiencing obstetric fistula have lost a child in the process, and may face reduced value and opportunity in cultures where social status is dependent upon childbearing [18, 19]. These women may or may not be able to have another child, which will determine their future opportunities. Above all, it is impossible to erase the experience of obstetric fistula, regardless of their new physical status after repair. Women returning from obstetric fistula repair may be returning to an environment, which has been hostile to them, and it is unknown to what extent reintegration parallels pre-fistula status.

The few studies that have evaluated the post-repair reintegration experience have reported improvement in perceived quality of life, particularly among women with successful surgery [20, 21]. A qualitative study in Tanzania revealed that most women were able to resume their household and farming responsibilities after the fistula repair, and felt that their ability to resume working was the most important factor for success of their reintegration process [20]. These women reported significantly higher perceived quality of life and resumption of normal living post-childbirth after surgery, when compared to living with the fistula. In this and other studies, women with lingering physical problems such as urinary stress incontinence, pain and fatigue were less able to resume their previous roles and were less likely to consider themselves recovered [7, 20–22]. Quality of life improvement post-repair seems to be linked to length of time lived with fistula; one study found greater improvements in quality of life among women who lived with fistula for greater than one year compared to those women who were more swiftly repaired [21]. The reintegration experience is much improved with family support, particularly given economic and emotional needs [20]. While most women return to their families, some choose not to return to their previous residence [23, 24]. Across many studies women were divorced from their husbands after fistula, although this varies significantly by geographic location and presence of children [21, 23]. Many women expressed providing for themselves as a continuing concern after fistula repair, particularly those that were no longer married or had less family support [24]. In addition to pure economic survival, women reported that having work and being able to provide for themselves was a way to restore their value as a women [24].

Furthering the evidence base around reintegration experiences after obstetric fistula surgery and understanding the effect of interventions to enhance the reintegration experience requires an explicit attention to measurement. Currently no standardized measurement tool exists to evaluate how successful a woman’s post-surgical reintegration has been. Therefore, the primary goals of our research project are to understand the process of family and community reintegration post fistula surgery; and to develop and pilot test a tool to assess the long-term success of family and community reintegration among women after obstetric fistula surgery. The development of such a tool has important implications for development and evaluation of evidence-based programming for the growing population of women with fistula repair.

This study is currently in progress. Our formative qualitative work and instrument development have been completed (June–August 2014). Instrument validation within our longitudinal cohort is ongoing (November 2014–present). This paper presents the full protocol for the project activities including the qualitative component, instrument development and validation.

## Methods

### Study design

An exploratory sequential mixed-methods design was chosen for the study given the limited literature on the topic of obstetric fistula reintegration [25]. We plan to conduct initial qualitative research (in-depth interviews followed by focus group discussions) among women who had previously had obstetric fistula surgery to explore and confirm the domains relevant to reintegration among this particular patient population. The identified domains will be used to develop the standardized reintegration measurement tool. A quantitative longitudinal component will establish the reliability and validity of the measurement tool, with a 12-month follow up enabling us to evaluate the reintegration process for a lengthier period of time than what has been previously observed. This longitudinal component will also contribute to our understanding around the individual and other characteristics that contribute to successful reintegration. Although it was not part of the originally funded protocol, the research team implemented a nested medical record abstraction component for all records of patients managed within the fistula care unit from 2005 onward to enable the evaluation of hospital-specific surgical outcomes and predictors of successful surgical outcome at the study site, after approval by both institutional review boards.

### Study population

The target population for our study is women accessing obstetric fistula repair services at Mulago Hospital in Kampala, Uganda. Mulago Hospital is the national referral hospital and the teaching hospital for Makerere University School of Medicine. Fistula repair is provided by the urogynecology division as both an ongoing surgical service and supplemented by four to five one-week long fistula camps conducted annually.

Eligibility for participation in the qualitative component is defined as having undergone obstetric fistula surgery within the previous 6–24 months at Mulago Hospital, speaking Luganda or English, residing within 100 km of Mulago Hospital, providing a telephone contact at surgery, and ability to provide informed consent for study participation. Eligibility for participation in the longitudinal component is defined as completion of initial examination and clearance for obstetric fistula surgery at Mulago, speaking Luganda or English, residing in a community with cellular coverage, and ability to provide informed consent for study participation.

### Sample size

We anticipate enrolling a total of 120 individuals in the study overall, including both qualitative and quantitative components, based on the nature of the data collection, budget and proposed time period. Our final qualitative sample will be determined by the point at which we achieve data saturation; we anticipate conducting in-depth interviews with approximately 15 women followed by focus group discussions among 24–32 women. We plan to enroll 60 women within our longitudinal cohort from the time that they access fistula surgery through 12 months post-surgery. As this is an exploratory study, we have based our sample size on the number of participants we can feasibly include given the estimated number of surgeries to be conducted over the study timeline and the resource constraints of the project. We anticipate that approximately 2000 medical record abstractions will be completed.

### Participant recruitment

Potential study participants for the qualitative component will be identified via urogynecology division surgical logbooks, which list patient name, medical record number, location of residence, next of kin including contact number, surgery conducted and surgical outcome. The research staff will abstract the first name, medical record number, contact number, and surgical outcome of all individuals who underwent obstetric fistula surgery within the previous 6–24 months, provided a telephone contact number and live within 100 km of Mulago Hospital based on their provided location of residence. The research staff will then contact these individuals by telephone, screen them for eligibility, explain the purpose of the study to them, and obtain verbal consent from those who indicate an interest in participating. Women who verbally consent to participate will be scheduled for either an in-depth interview or focus group discussion at Mulago Hospital. When participants arrive at Mulago Hospital at the scheduled time for study participation, the details of the study will be re-explained to them and written informed consent will be obtained via signature or thumbprint, prior to interview or focus group. The consent form and other study documents will be available in Luganda and English, and interaction with the research staff will occur in whichever language the participant indicates they are most comfortable in. Participants will be reimbursed for their round-trip transportation expenses from their residence to Mulago Hospital and will be provided with refreshments during their participation.

Potential study participants for the longitudinal cohort will be identified in the urogynecology inpatient admission logbook. Admitted patients confirmed to have obstetric fistula will be approached and screened for eligibility by the research staff, who will present the details of the study and request their participation. The research staff will then obtain their written informed consent via signature or thumbprint from those who are interested. Participants who are unable to be approached prior to surgery will be asked to participate after they have undergone surgery and are sufficiently recovered to converse with the research staff. The consent form and other study documents will be available in Luganda and English, and interaction with the research staff will occur in whichever language the participant is most comfortable in.

### Data collection

#### Qualitative data collection

Data for our qualitative study participants will be obtained from demographic questionnaire, in-depth interview or focus group, and medical record abstraction.

Prior to their participation in in-depth interviews or focus groups, the qualitative participants will be asked to complete a short demographic questionnaire to capture the following characteristics: age, tribe, religion, household characteristics, marital status and history, employment and financial status, educational attainment, pregnancy and obstetric history, general health and level of continence, and receipt of post-surgical counseling. Continence will be evaluated with the International Consultation on Incontinence Questionnaire Short Form (ICIQ-SF) which has been validated with objective leakage measurements [26]. General health will be evaluated with the Stanford Self-Rated Health measure [27].

Women participating in in-depth interviews will be asked open-ended questions about their life and health prior to development of the fistula, the pregnancy and labor leading to the fistula, life with the fistula, treatment seeking and reintegration experiences. To explore the women’s experience with reintegration into family and community life, women will be asked specifically about the reaction of her husband and family upon her return, her experience resuming regular household duties, and how she was able to reintegrate into her community (e.g., participation in social and religious activities, and income-generating activities, etc.). The in-depth interviews will also focus on resumption of sexual activity since obstetric fistula surgery and fertility intentions.

Focus group discussions will be more targeted to the domains that emerge as most important to reintegration that are identified in the in-depth individuals in order to inform the reintegration tool development.

In-depth interviews and focus group discussions will be audio recorded with the explicit permission of participants, and the data will not include names or other potentially identifying information. Audio files will be concurrently translated (if in Luganda) and transcribed by an experienced bilingual transcriptionist shortly after recording. Another translator will randomly select transcripts to assess the quality of the translation and transcription.

#### Quantitative data collection

Data for our longitudinal cohort participants will be obtained from a series of surveys collected from confirmation of obstetric fistula survey through 12 months post-surgery and from medical record abstraction. Data will be collected at baseline, 2 weeks, 3 months, 6 months, 9 months, and 12 months post-surgery.

The baseline questionnaire will include basic socio-demographic questions, the newly developed/modified measurement tool, and mental health, quality of life, self-esteem, trauma and social support measures. We will use the following validated measurement tools: the International Consultation on Incontinence Questionnaire Short Form (ICIQ-SF) [26], the Stanford Self-Rated Health measure for general health [27], the Hopkins Symptom Checklist for depression [28, 29], the WHO QOL BREF for quality of life [30, 31], the Rosenberg self-esteem scale [32], a modified version of the HIV/AIDS Stigma Instrument [33], the Primary Care PTSD Screen and the Brief Trauma Questionnaire for trauma [34, 35], and the multidimensional scale of perceived social support [36, 37].

The follow-up surveys will include any modifiable socio-demographic questions, the newly developed reintegration measurement tool, any further questions on reintegration which arise as necessary to address specific issues mentioned as primary challenges to living with obstetric fistula in the Ugandan context, and the validated mental and physical health instruments listed above. The reintegration tool will be readministered prior to hospital discharge at approximately 10–14 days after the baseline survey for evaluation of temporal stability of the tool. 

All four post-discharge follow-up surveys will be administered over mobile telephone by the research staff. Participants will be provided with cell phones and monthly airtime contracts for the duration of the follow-up. Any participants that already have a cell phone are not given another phone but will be given monthly airtime contracts for their own phone. At the end of each call the participants will be asked several questions about their perspective of reporting on these experiences on a call versus in person; we will track the number of call attempts per data point per person, and participant and research staff perspectives on the ease of mobile data collection.

Medical records will be abstracted for data on fistula classification, surgical outcome, and any other relevant clinical information using a standardized abstraction form, excluding personally identifying information.

### Data analysis

#### Qualitative data analysis

Transcripts from in-depth interviews and focus group discussions will be coded using inductive and deductive codes within Atlas.ti software and analyzed for domains most relevant to family and community integration. Thematic analysis will occur concurrent with data collection to assist in determining achievement of data saturation. The qualitative results will be used to inform the development of the reintegration instrument in conjunction with a review of the literature on measurement of reintegration after experience of comparable conditions. Based on our preliminary research, the study team anticipates that the tool will follow the format of the Return to Normal Living Index (RLNI) [38], an 11-item scale that has been translated and validated among a variety of populations, and is commonly used among victims of stroke and other trauma to assess the degree to which an individual feels normalized in their environment. We will modify the tool and add questions as necessary to reflect the Ugandan women’s domains of interest and to ensure the tool’s relevance to the context and lives of Ugandan women. These decisions will be made based on the qualitative results. Once the pilot tool is formulated we will follow the WHO recommended process for translation and adaptation of instruments: forward translation, expert panel back translation, pre-testing and cognitive interviewing, and finalizing the version [39].

The first stage of analysis will involve coding and classification of the data by reviewing the transcriptions for potential conceptual categories, using the guideline questions as initial categories. Two types of codes will be employed. Firstly, deductive codes that represent expected influences on the outcomes will be applied to the data: these will be taken from the existing literature on obstetric literature and repair and our interview and focus group guides. Secondly, inductive codes that emerge organically from the data will be applied: these codes represent themes that are not expected by the researchers. Emergent themes will be identified based on recurrence rate and on similarities and differences noted across the texts. A codebook will be developed from the themes and included a detailed description of each code, inclusion and exclusion criteria, and examples of the code in use. Coded data will be analyzed thematically to describe the different dimensions and commonalities of each theme, their distribution across demographic variables, degree of fistula and clinical symptoms, experiences since fistula care and treatment and reintegration into family life and community, and the patterns and linkages between themes. This will allow us to build concepts grounded in the data to explain phenomena. Comparisons will be made with the data to detect divergent views among the participants and to contrast observations that relate to variables within the sample population (e.g. variations by age, family composition, continence level, duration of living with a fistula, and exposure to fistula education and prevention efforts).

#### Quantitative data analysis

Our reintegration measurement tool will be pilot tested in the context of a small longitudinal cohort of women accessing obstetric fistula surgery at Mulago Hospital. Quantitative data collection will occur at baseline, prior to hospital discharge and 3, 6, 9 and 12 months post-surgery. Internal consistency reliability will be evaluated at each data collection point while temporal stability reliability will be evaluated at baseline (hospital entrance to exit, approximately 10–14 days). Content validity will be established through key informant review. Construct validity will be examined through known-groups validation by several demographic and functional variables. Confirmatory factor analysis will verify the latent variable structure of the scale. The socio-demographic characteristics and clinical outcomes of the women will be described using one-way frequency tables as well as means, medians, ranges and standard deviations for the reintegration tool. The distribution of the reintegration tool will be compared by severity of fistula, perceived success of fistula surgery, initial clinical success of surgery, exposure to fistula education and prevention efforts, clinical services and activities, age, parity and socio-economic status. Differences will be tested following distributional assessment utilizing chi-square tests, t-tests, and/or Wilcoxon rank-sum tests. To examine the internal consistency reliability of the scale, Cronbach’s α statistic will be calculated at each data collection interval. Pearson’s correlation coefficient or Spearman’s rho will be used to examine the degree of correlation between two baseline measures (hospital entry and hospital exit) among a subset of the longitudinal sample to assess temporal stability reliability. Construct validity assessment will evaluate the degree of association between the tool scores and demographic variables (e.g., age, size of fistula, duration of fistula, incontinence severity measure score) through ANOVA or regression analysis. Finally, confirmatory factor analysis will be used to confirm the number of latent variables underlying the scale utilizing an orthogonal rotation.

The demographic characteristics, fistula characteristics and clinical outcomes of the women whose medical records have been abstracted will first be described using one-way frequency tables. Bivariate analyses will be conducted to evaluate the relationship between demographic, fistula and surgical characteristics of patients and surgical success. Differences will be tested following distributional assessment utilizing chi-square tests, t-tests, and/or Wilcoxon rank-sum tests.

## Discussion

This exploratory sequential mixed-methods study will provide information regarding the success of family and community reintegration among women returning home after obstetric fistula surgery in Uganda. Our study will provide the clinical and research community with a measurement tool for standardizing measurement across this patient population. In addition, our project will also inform feasibility of data collection via mobile phone for this patient population, which will be very useful for conducting research within similar populations. Due to the exploratory nature and small sample size of our study, our results will not be generalizable; however, a larger multi-site follow-up study evaluating the measurement tool is planned. Our evaluation of surgical outcomes and predictors of successful surgery will assist the uro-gynecology division in tracking outcomes and implementing best practices. The results of the study will be presented at international conferences and published in peer-reviewed journals, and we hope to utilize our results to inform intervention programing to improve women’s experiences with obstetric fistula.

## Ethical approval

The study protocol was approved by the Makerere University School of Medicine, College of Health Sciences Research Ethics Committee (Ref# 2014–052) and the University of California, San Francisco Human Research Protection Program, Committee on Human Research (IRB# 12–09573). All individuals eligible for the research will undergo an informed consent process; those individuals unable to provide signature for informed consent will provide thumbprint confirmation.

## Abbreviations

OF: obstetric fistula
